# Preservation of protein fluorescence in embedded human dendritic cells for targeted 3D light and electron microscopy

**DOI:** 10.1111/jmi.12230

**Published:** 2015-03-18

**Authors:** K. HÖHN, J. FUCHS, A. FRÖBER, R. KIRMSE, B. GLASS, M. ANDERS‐ÖSSWEIN, P. WALTHER, H.‐G. KRÄUSSLICH, C. DIETRICH

**Affiliations:** ^1^ Department of Infectious Diseases, VirologyUniversity Hospital HeidelbergHeidelbergGermany; ^2^ Carl Zeiss AGOberkochenGermany; ^3^ Carl Zeiss Microscopy GmbHJenaGermany; ^4^ Electron Microscopy FacilityUlm UniversityUlmGermany

**Keywords:** Confocal laser scanning microscopy, correlative light and electron microscopy, fluorescence preservation of embedded samples, focused ion beam, targeted FIB

## Abstract

In this study, we present a correlative microscopy workflow to combine detailed 3D fluorescence light microscopy data with ultrastructural information gained by 3D focused ion beam assisted scanning electron microscopy. The workflow is based on an optimized high pressure freezing/freeze substitution protocol that preserves good ultrastructural detail along with retaining the fluorescence signal in the resin embedded specimens. Consequently, cellular structures of interest can readily be identified and imaged by state of the art 3D confocal fluorescence microscopy and are precisely referenced with respect to an imprinted coordinate system on the surface of the resin block. This allows precise guidance of the focused ion beam assisted scanning electron microscopy and limits the volume to be imaged to the structure of interest. This, in turn, minimizes the total acquisition time necessary to conduct the time consuming ultrastructural scanning electron microscope imaging while eliminating the risk to miss parts of the target structure. We illustrate the value of this workflow for targeting virus compartments, which are formed in HIV‐pulsed mature human dendritic cells.

## Lay Description

Confocal laser scanning microscopy and focused ion beam scanning electron microscopy are two established techniques to obtain three‐dimensional microscopic data of specimen. Since they are based on different interaction mechanism of photons or electrons, they provide complementary information on function and morphology of a sample. Their combination is desirable but difficult since in general sample preparation protocols are different or even exclude each other. We present a workflow for suspension cells, which includes a sample preparation to comply with both imaging methods. Furthermore, a microstructured reference coordinate system is imprinted at the specimen's surface, which is visible for both instruments and serves as a common reference. The specimen is mounted and oriented on a three‐dimensional sample holder to conveniently transfer the sample between both instruments. confocal laser scanning microscopy is used to identify and image target regions, which are revisited and imaged with the electron microscope afterwards. We successfully employed the workflow for virus compartments, formed in HIV‐pulsed cells. The preservation of virus fluorescence during preparation allowed to identify compartments with confocal laser scanning microscopy and to obtain target coordinates with micrometre precision. This allowed accurate guidance of the focused ion beam scanning electron microscopy to resolve ultrastructural details of the compartment.

## Introduction

Life science research increasingly demands multimodal imaging methods to obtain comprehensive data about dynamic events. Light microscopy (LM) provides cellular localization and distribution patterns of genetically labelled proteins, but lacks information about ultrastructural details. A prospect in modern microscopy is the potential to augment live cell fluorescent imaging with direct high resolution ultrastructural information provided by electron microscopy (EM) (Biel *et al*., 2003; Pelletier *et al*., 2006). Such correlative light‐ and electron microscopic techniques rely on different localization strategies. One possibility is to identify cells transiently expressing genetically encoded fluorophores prior to fixation that guide the EM ultrastructural analysis of the specimen (Müller‐Reichert *et al*., 2007; Kolotuev *et al*., 2009; Guizetti *et al*., 2011). Other correlative microscopic methods identify specific fluorescent patterns directly in the EM sample after preparation (Sartori *et al*., 2007; Nixon *et al*., 2009; Watanabe *et al*., 2011). Such preserved fluorescent protein signals in high pressure frozen EM samples could directly be correlated with three‐dimensional (3D) electron tomographic data as shown by Kukulski *et al*. in 2011. In this study, the preserved GFP signals allowed the targeting of ultrastructural features like HIV particles and microtubule end structures with high precision in 300 nm Lowicryl sections. Such approaches allow elucidating suborganelle‐sized structures that are small enough to be captured in thick plastic sections, but visualizing structures within a bulky cellular volume that extend beyond a single or even a series of thick sections is difficult and requires a major effort.

A novel imaging technique for acquiring high resolution 3D data of bulky biological tissue is focused ion beam scanning electron microscopy (FIB‐SEM). This method utilizes a SEM equipped with a gallium ion source to produce a second FIB. Both beams coincide at one point in the microscope where the sample is located and can therefore be used to image the same area without moving the microscope stage under the two beams (Heymann *et al*., 2006; Hekking *et al*., 2009 and others). Furthermore the ion beam can be used to remove a few nanometres of the sample surface thus exposing the freshly generated block‐face that is then imaged with the electron beam in an iterative manner. This procedure can then be continued and a stack of two‐dimensional images is generated. By reconstructing the imaged volume, a 3D high resolution representation of several micrometres of the biological material is produced (Knott *et al*., 2011). FIB‐SEM approaches on high pressure frozen and resin‐embedded samples have already proven that good ultrastructural data strongly depend on EM preparation protocols that preserve the fine ultrastructural details in the cell and more importantly that allow a strong heavy metal staining of membranes in the resin (Villinger *et al*., 2012).

Typically only specific subcellular areas within a large volume of resin are of interest. Therefore, a preselection of regions prior to the FIB‐SEM procedure becomes necessary to efficiently target the volume(s) of interest (VOI). Correlative microscopy enables the identification of sometimes rare features of interest on a larger scale and afterwards navigation of the FIB‐SEM to the target area (Lucas *et al*., 2012).

The correlative system introduced in this work was applied to study the biologically significant interaction between mature human dendritic cells and HIV‐1. Dendritic cells are potent antigen‐presenting cells and play a unique role in initiating the primary immune response. HIV has developed strategies to subvert the antiviral activity of the immune system. Virions specifically bind SIGLEC‐1 on the mature dendritic cell (mDC) surface and are sequestered and stored in these cells via a noninfectious pathway (Izquieros‐Useros *et al*., 2014). mDCs are generally not productively infected with HIV‐1, but are able to transfer virus to susceptible T‐cells in a process called *trans*‐infection (Izquieros‐Useros *et al*., 2014). Insights into the mechanisms of virus sequestration has come from light microscopic analysis revealing captured virus accumulating in large, discrete intracellular sac‐like compartments (Izquieros‐Useros *et al*., 2012). Other studies utilized FIB‐SEM to explore the 3D architecture of accumulations of HIV virions in deep membrane‐enclosed reservoirs in macrophages at higher magnification (Bennett *et al*., 2009). Correlative light and FIB‐SEM imaging has also been used to analyse the localization of HIV particles in mammalian cells (Murphy *et al*., 2011) and to investigate cell‐to‐cell contact regions between HIV‐infected T cells and several types of uninfected cells, including primary human CD+ T cells and primary human foetal astrocytes (Do *et al*., 2014). They investigated chemically fixed and epoxy resin embedded cells with strong heavy metal stained membranes. In this case, the cell contact regions of interest were identified by fluorescence wide field LM before EM sample preparation.

Here, we present a novel correlative microscopy solution that combines the possibility to localize fluorescent proteins with confocal microscopy in 3D and uses this information to guide the FIB‐SEM to the respective VOI in 3D with high precision. The workflow starts with fluorescence live cell microscopy of virus‐like particles captured by mDCs. An optimized specimen preparation protocol based on high pressure freezing was developed that allows preserving the fluorescent signal after freeze‐substitution and resin embedding along with a good ultrastructural preservation. This allowed identification of dense viral compartments by confocal laser scanning microscopy and their subsequent high‐resolution FIB‐SEM imaging. A coordinate system on the surface of the resin block was used as a reference that enables the relocation of the respective structures of interest with a high spatial precision and a robust and precise image correlation between different imaging modes.

## Material and methods

### Primary cell cultures

Peripheral blood mononuclear cells were isolated from blood of HIV‐1‐seronegative donors as previously described (Izquierdo‐Useros *et al*., 2012). Monocyte populations were obtained with CD14+‐positive selection magnetic beads (Miltenyi Biotec, Bergisch Gladbach, Germany). CD14+ populations were cultivated in presence of 1000 IU mL^−1^ of granulocyte‐macrophage colony‐stimulating factor and interleukin‐4 (R&D, Minneapolis, MN, USA) to differentiate them into immature dendritic cells. Maturation of DCs was induced by culturing immature dendritic cells at day five for two more days in the presence of 100 ng mL^−1^ lipopolysaccharide (LPS; Sigma‐Aldrich, Steinheim, Germany).

### Isolation of HIV‐1

HIV‐1 virus‐like particles fluorescently labelled by mCherry inserted into the structural Gag polyprotein (VLP_HIV‐Gag‐mCherry_) were produced by transfection of HEK 293 cells with pCHIV MAmCherry using calcium phosphate. Tissue culture supernatant was harvested 48 h after transfection. Particles were enriched and partly purified by ultracentrifugation (100 000 g for 2 h) through a cushion of sucrose. Concentrated particles in the pellet were resuspended in PBS and quantified by Western plotting detecting the viral capsid protein.

### Sample preparation EM

mDCs were pulsed with 50 ng HIV‐1‐ VLPs_HIV‐Gag‐mCherry_ for 5.5 h and placed on glow‐discharged, carbon‐coated and Poly‐L‐Lysin (Sigma‐Aldrich) covered 160 μm thick sapphire discs (Engineering Office M. Wohlwend GmbH, Sennwald, Switzerland). After attaching onto the sapphire discs, cells were subsequently high pressure frozen (HPM 010, Bal‐Tec, Balzers, Lichtenstein). A coordinate system is imprinted on the surface to facilitate orientation on the sapphire discs and to provide a reference for different imaging modes. The samples were further processed by freeze substitution in a temperature‐controlling device (AFS2, Leica, Wetzlar, Germany) with a substitution medium consisting of acetone supplemented with 0.1% uranyl acetate and 5% water. The temperature was gradually raised from −90 to −20 °C over a 16 h period. After washing the samples three times with 100% acetone at −20 °C, the cells were infiltrated with increasing concentrations (25, 50, 75 and 100%; 1 h each) of LR‐Gold (London Resin Company, Reading, England) in acetone. The final embedding in 100% LR‐Gold occurred over 12 h and samples were UV polymerized starting at −20 °C for 24 h, after which the temperature was raised to 20 °C over 4 h and UV polymerization continued for 24 h.

### Light microscopy

For 3D fluorescence LM we used a laser scanning microscope (LSM710 Carl Zeiss Microscopy GmbH, Jena, Germany). We built a block holder (Fig. S1), which allows free 3D rotation of the block to achieve an orientation of the block surface perpendicular to the optical axis of the microscope. Therefore, the resin block was fixed in the hole, drilled through the centre of a plastic sphere. The sphere freely glides in the bearing of opposing cylinders to allow free orientation of the block. With a simple leverage mechanism, one cylinder was moved toward the sphere and locks it in a desired orientation. For a rough alignment we employed a simple low NA objective in epi illumination configuration (5×/0.13 A‐Plan) to optimize the reflection signal from the block surface. For fine tuning, the objective was replaced by a standard glass slide, which was placed on the empty objective position of the objective turret. Epi‐illumination light is reflected at the glass slide and at the surface of the sample block, which results in two images of the field stop, observable with the eyepieces. The lateral position of the field stop in the image resulting from the reflection at the glass slide is stationary and serves as the reference for the optical axis of the imaging system. The block was then oriented until both reflection images coincided. This ensured that the images recorded with the microscope were well aligned to the coordinate system imprinted into the surface of the resin block.

For imaging, we found that oil immersion objectives with high NA are very suitable, since the refractive index of the resin matches well with this immersion. For LM we kept the sapphire disc as a protective layer on the resin block to avoid pollution or degradation of the resin block for later SEM imaging. Reference marks at the interface were easily imaged in the confocal reflection mode, which we typically recorded as a z‐stack for a whole coordinate field of 200 × 200 μm. The axial position and tilt of the block‐face was accurately determined by the location of the maximal intensity of the reflection signal within the z‐stack. After the alignment process described above, we found the maximum measured tilts to be consistently below 1°, i.e. the offset in axial direction within a field of 200 × 200 μm was less than 5 μm. In consequence, for objects buried 40 μm below the surface it is sufficient to account for an additional paraxial error of less than 1 μm. At this point, any stage position was locked and further overview or detail imaging was conducted by mere control of the scanners of the confocal microscope. For detail imaging the crop function was employed, to record interesting regions with the desired resolution and contrast mode. This ensured that uncertainties of lateral voxel positions of all recorded image data were in the range of positioning error of the scanners, which is well below the pixel resolution of recorded images, i.e. << 1 μm. As a result the LM data now reflect correctly the location of target structures in the bulk volume with respect to the coordinate marks at the surface of the resin block.

### FIB milling and SEM imaging

Removing the protective sapphire disc from the resin block was performed by bringing the block sample close to the surface of liquid nitrogen. Any direct contact between resin and liquid nitrogen has to be avoided, since this can cause the formation of cracks in the resin, compromising the integrity of the sample. The process was repeated, until the sapphire disc could be removed with tweezers, without the need to apply any significant force.

All surfaces except the tops of the resin blocks were covered with silver paint to ensure good conductivity and avoid charging. The top surface with the imprinted marker grid was coated with a 10–20 nm thick palladium layer in a sputter coater (Sputter Coater 208Hr, Cressington Scientific, Watford, England, United Kingdom) to facilitate charge dissipation. For 3D EM imaging, the sample was placed in a FIB‐SEM (Crossbeam Neon40, Carl Zeiss Microscopy GmbH, Oberkochen, Germany). The target VOI was relocated in two steps. First, the imprinted coordinate system was utilized to find the grid position again that contained the previously acquired VOI. Secondly, the surface image of the grid square was correlated with the reflection image from the confocal microscope as described in detail in the section experimental results. Afterwards an additional protective platinum layer was deposited directly on top of the target volume using ion beam assisted platinum deposition with 30 kV acceleration potential. A coarse cross‐section was milled to get access to the targeted structure and as a viewing channel for SEM observation using 10 nA ion beam current. The exposed surface of this cross‐section was polished with a beam current of 2 nA. Subsequently, 25 nm thick layers were removed with the ion beam alternated with SEM imaging of the freshly exposed surface. SEM images were acquired with 2.8 keV acceleration potential and a 30 μm aperture using a secondary electron detector. The images were obtained with size of 3072 × 2304 pixels at a resolution of 6.4 nm per pixel.

### Image processing and segmentation

For alignment of scanning electron micrographs from consecutive FIB‐SEM sections a feature‐based method was applied in a similar fashion as described by Saalfeld *et al*. (Saalfeld *et al*., 2010). Successive images were stacked with the open source software ImageJ. Manual segmentation of the dataset was performed using the Amira 5 software (FEI, Visualization Sciences Group, Eindhoven, Netherlands). The 3D Maximum intensity projection with combined segmentation was performed using ORS (ORS, Visual SI, Canada).

### Experimental results

**Figure 1:**
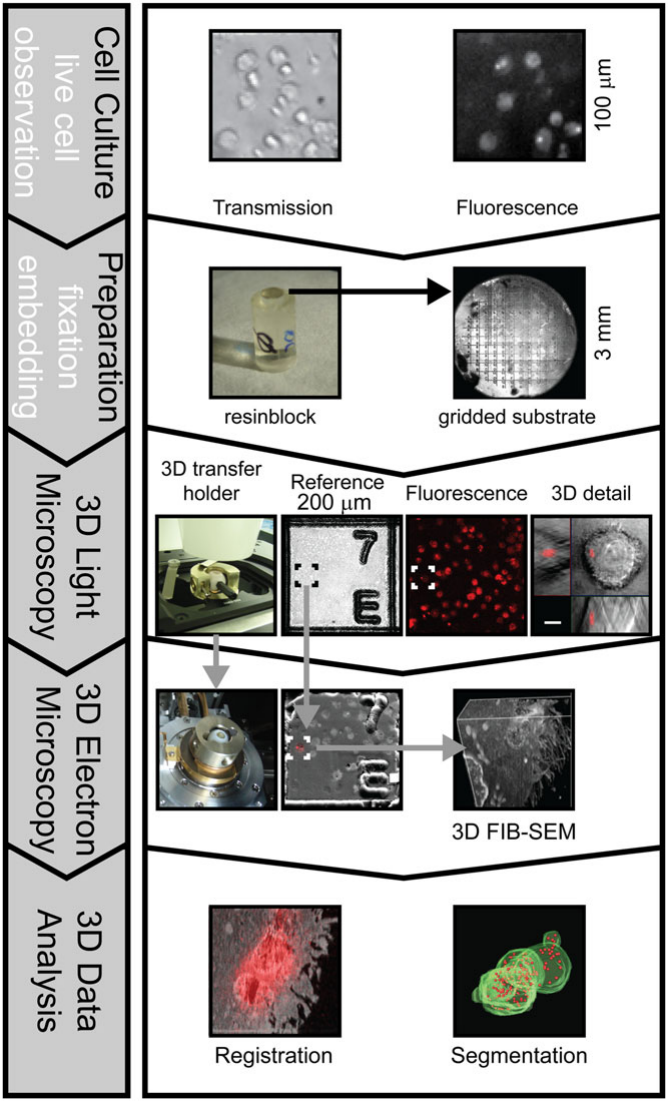
Generalized scheme of the correlative 3D workflow. Since fluorescence is preserved during the EM preparation process, 3D confocal fluorescence microscopy allows selection and detailed imaging of target structures within the resin block. Additional recording of coordinate marks on the block surface allows the transfer of target coordinates to guide FIB‐SEM imaging with micrometre precision. Defined orientation of the block surface is ensured by the 3D transfer holder, which allows to align the block surface perpendicular to the z‐axis of the light microscope and to transfer directly the sample to the electron microscope. Exact guidance of FIB‐SEM by 3D light microscopy data restricts FIB‐milling and SEM imaging to target structures, minimizing processing time and data collection. Finally this simplifies the task to register light and electron microscopic datasets.

Our correlative microscopy workflow aims at localizing subcellular structures of interest in plastic embedded samples. Its foundation is the preservation of fluorescent signals in the resin after EM preparation, which allows identification and precise localization of target structures, i.e. VOIs. This in turn allows the precise guidance of the FIB milling to selected cellular structures. A general scheme of the workflow is depicted in Figure 1.

Live cell imaging was important to monitor the expression of transgenic proteins. In our case the fluorescence signal was used to identify the distribution pattern of mCherry labelled virus‐like particles captured by human mDCs. To be able to control the amount of mDCs that internalized virus‐like particles in the fluorescence microscope immediately prior to fixation, cells in a small suspension volume were plated onto sapphire discs with an engraved coordinate system on the surface. This sapphire discs serve as gridded substrates that ensure a stable and accurate reference at the block surface after the EM preparation.

For fluorescence preserving preparation, the cells on the gridded sapphire discs were high pressure frozen, freeze substituted and finally embedded in LR‐Gold resin using an adapted protocol introduced by Krisp *et al*. (2007). After the polymerization, the coordinate system of the sapphire disc leaves an imprint on the surface of the polymerized resin block that serves as an accurate and stable reference system. The protocol had to be optimized to preserve the fluorescence for LM in the freeze substitution and embedding procedure while conserving at the same time the fine cellular ultrastructural details with sufficient staining to enable FIB‐SEM imaging.

**Figure 2:**
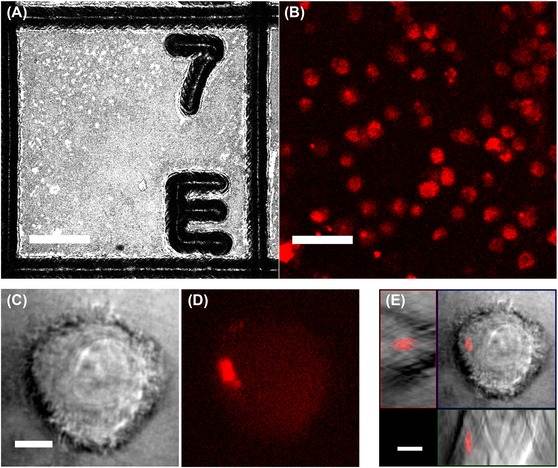
3D confocal light microscopy for identification and imaging of target structures. (A, B): Different contrast modes of laser scanning provide complimentary information of the block sample: A: Confocal reflection mode provides an accurate representation of the interface between the gridded sapphire disc and LR‐Gold resin. The plane within the z‐stack with the highest reflection signal indicates the position of the block surface. Since this maximal intensity is attributed to only one image of the z‐stack, it confirms the excellent alignment of the block sample achieved by the 3D block holder. Note, with this contrast cells in the block are not visible. B: Fluorescence image recorded below the block surface allows to identify cells which exhibit the desired fluorescence pattern. Frames in A and B indicate the lateral position of a cell with a condensed virus‐like particles containing compartment that was selected for imaging in more detail, shown in C–E. C: Transmission contrast is provided by laser light which passes the sample and which is then collected by a sensor. This contrast features no optical sectioning, since a pinhole is missing, but provides an additional morphological contrast to assess the state of the cells. D: Corresponding plane of fluorescence contrast shows a condensed fluorescence spot which indicates the formation of a dense virus compartment. E: The orthogonal view of the complete z‐stack gives a clear vision of the position of the virus compartment within the selected cell. All images were recorded with 63x objective lens (63x/1.4 Oil Plan Apochromat). Scale bars in (A, B) represent 50 μm, in (C–E) 5 μm.

For 3D LM imaging, a custom designed block holder (Fig. S1) allowed aligning the block surface orthogonal to the optical axis of the light microscope (see Material and Methods). This ensured a defined orientation of the recorded image stacks. Selection, referencing and detail imaging of target regions was performed with an immersion lens (63x/1.4 oil Plan Apochromat, Carl Zeiss Microscopy GmbH) by employing the precise and flexible laser steering features of the confocal microscope. An example for a dataset which demonstrates the imaging conditions as well the preservation of specific fluorescence is presented in Figure 2. A z‐stack with confocal reflection contrast allows to accurately localize the axial position of the block surface and gives a precise representation of the imprinted microstructures (Fig. 2A). Subsequent, fluorescence imaging with an open pinhole allows the effective imaging and selection of target structures in the bulk (Fig. 2B). For further detail recordings, the pixel resolution is then optimized and the field of view is restricted to regions of interest. Figures 2(C–E) shows detail recordings of a cell that was selected because it contained a condensed virus‐like particles carrying compartment. It is important to note that any lateral stage movement was omitted, since this would cause positioning inaccuracies. The resolution of the light microscope permits localizing subcellular structures, but is not sufficient to resolve details of the ultrastructural arrangement of the virus compartment.

**Figure 3:**
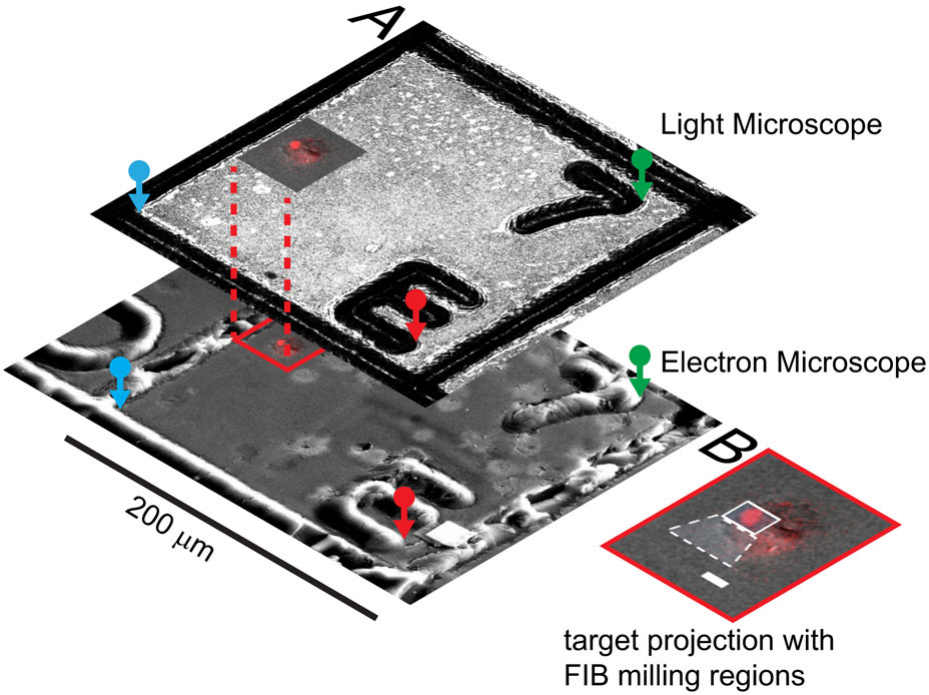
Precise transfer of target coordinates between light and electron microscope. Three corresponding pairs (red, green, blue) of reference points are manually marked in the two reference images (A). This allows calculation of the affine transfer function, which projects the fluorescence signal collected at the target site onto the surface image recorded with the FIB‐SEM microscope. Inset (B) shows the 3D fluorescence dataset projected onto the SEM surface image. The white rectangles indicate the windows selected for FIB‐milling. Scale bar represents 5 μm.

Accurate guidance of the FIB‐SEM is necessary, to collect data with nanometre resolution for the selected compartment and its local microenvironment. After attaching the SEM stage dove‐tail adapter, the 3D transfer holder was placed in the FIB‐SEM keeping the defined orientation of the block surface. Inspection of the sample with the SEM clearly showed the imprinted grid on the sample surface. The grid square that contains the cells of interest can easily be identified. The precise navigation to subcellular structures is achieved as illustrated in Figure 3(A). Three corresponding reference marks are defined in the LM and EM images of the reference layer. Their coordinates allow the calculation of the transfer function, which was used to project the fluorescence signal of the detail images onto the FIB‐SEM surface image. This process was performed in a semiautomated fashion using a custom built plug‐in for the LSM software (Carl Zeiss Microscopy GmbH). Afterwards, the window for milling and imaging the compartment structures can readily be set as shown in Figure 3(B). A high accuracy of the overlay enables the user to precisely select the volume that contains the subcellular target structure. Since the block surface was aligned to the optical axis of the LSM, this holds true as well for targets buried several tens of micrometres within the block. As a result 3D FIB‐SEM acquisition could be performed with a smaller tolerance region without the risk to miss the targeted structure. To test this, we embedded FITC‐marked melamine micro particles (Sigma‐Aldrich, St. Louis, MO, USA) into epoxy resin. After LSM imaging the positions of beads were projected onto the FIB‐SEM surface image as described above. We then milled trenches next to the predicted boundary box of the individual bead positions using the FIB. Consistently, we found the particles between the trenches within a tolerance of 1 μm (Fig. S2) demonstrating a targeting accuracy of our approach to be ≤1μm.

**Figure 4:**
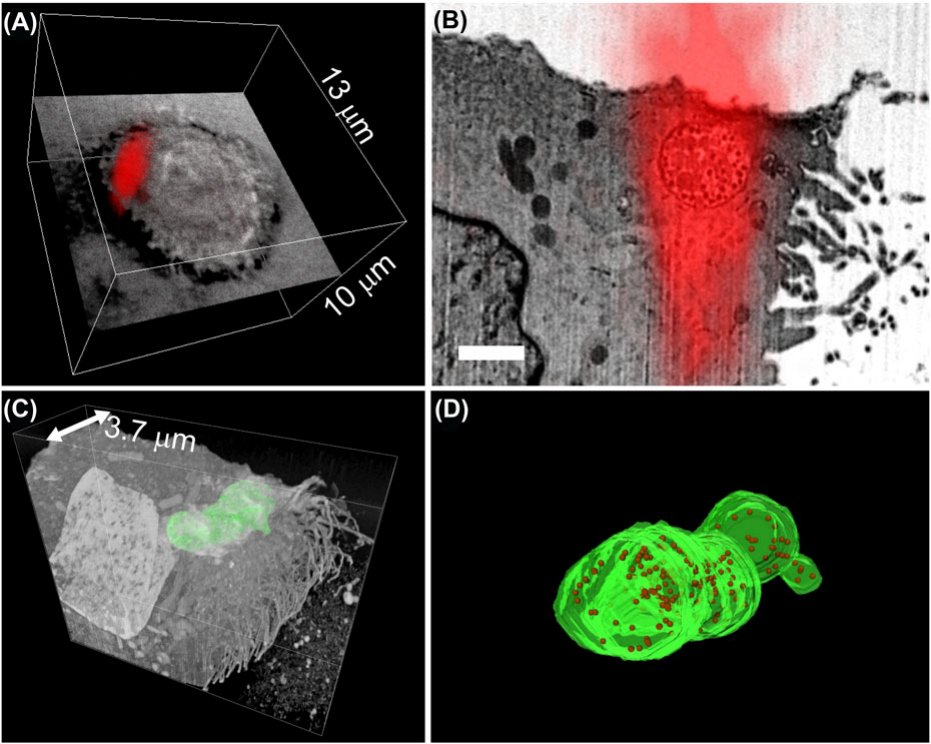
Combination and analysis of light and electron microscopy images recorded in the target region: A: 3D rendered fluorescence data set of the target cell. The fluorescence corresponds to the accumulation of internalized virus‐like particles. One layer of none confocal transmission contrast was included, to indicate the outline of the cell. B: Overlay of a single FIB‐SEM section with the corresponding section of the LSM image illustrates the correct guidance of FIB‐SEM imaging by functional fluorescence contrast. Reduced axial resolution of optical imaging results in a prolonged signal distribution along the z‐axis. Bar represents 1 μm. C: 3D FIB‐SEM dataset reveals the complete 3D structure of the virus‐like particles containing compartment (membrane is highlighted in green). D: Detailed view of the segmented compartment. The membrane is coloured in green and virus‐like particles are represented by red spheres.

Due to the precise guidance the sampling volume, the amount of gathered data and the acquisition time for the individual target sites can be minimized. Data were analysed and registered by state of the art image processing and visualization tools (Fig. 4). The biological results obtained for the dense virus compartments will be discussed elsewhere (publication in preparation).

## Conclusion

For the understanding of biological processes, the knowledge of temporal–spatial behaviour of processes in three dimensions is essential. The beneficial combination of functional and morphological information has led to a strong awareness and development of increasingly sophisticated correlative light and EM methods and protocols.

3D laser scanning microscopy and FIB‐SEM are well established techniques that deal with the imaging of 3D samples. A number of publications demonstrated correlative workflows which include fluorescence imaging prior to or following chemical fixation, heavy metal staining and resin embedding for FIB‐SEM imaging (Maco *et al*., 2014; Do *et al*., 2014). Such preparation procedures aim at generating good membrane visibility and yield good EM data, but do not preserve the fluorescence signal in the final sample used for FIB‐SEM. Since the investigated human dendritic cells are nonadhesive suspension cells, an accurate localization of cells in the light microscope prior to fixation is not possible. Mounting the sapphire disc between the aluminium planchets into the high pressure freezing holder causes significant disturbances. Cells can be identified, but can change their position or show overlapping fluorescent signals with other cells and thus prevent exact relocalization of cells for the targeted 3D EM imaging using FIB‐SEM. To overcome this, we applied an EM preparation protocol that preserves the fluorescence of proteins during the EM preparation steps and enables the localization of fluorescent structures in the resin block in the light microscope. In consequence, cells with different viral compartment patterns can be identified and selected for FIB‐SEM imaging, which provides detail imaging of viral cellular structures in 3D (see Fig. S3). For future applications, the here presented workflow can be adapted to be used for other resin embedded samples, e.g. Lowicryls, which have already been used in correlative studies (Nixon *et al*., 2009;Kukulski *et al*., 2011; Lucas *et al*. 2012).

Certainly reduced heavy metal staining causes less prominent visibility of some structures, e.g. membranes. At this point we could only partially compensate this, by increased detection times, which prolonged data acquisition. This might be overcome in the future, by further optimization of preparation and image acquisition parameters.

The workflow described here is tuned to enable the simple and defined transfer between both imaging modalities. The 3D transfer holder enables the defined orientation and convenient transfer of the sample between different devices (see Fig. S1). The microstructured reference layer at the block surface is essential for precise retrieval of target structures. We found the lateral dimension of 200 μm of the grid on the sapphire disc to be very convenient, since a low zoom parameter enables the imaging of a complete grid field with a 63× high NA objective lens (Fig. 2A). Further detail imaging is then very conveniently done by employing the scanning options of the 3D confocal laser scanning microscope.

In consequence, recorded 3D fluorescence data sets are in excellent correlation with FIB‐SEM data since intermediate sample processing is omitted, which could cause displacements, distortions or material variations. Indeed, a correlation of LM and EM data with high precision in the range of a few ten nanometres is only possible if the sample does not change between LM and EM imaging. Even the introduction of correlative fiducials cannot account for anisotropic shrinkage of tissue or the movement of suspension cells that are not fixed to the substrate. From that perspective it is desirable to further optimize the resolution of LM imaging in future studies, as indicated by the strong elongation of the fluorescence signal in axial direction (Fig. 4B). The removal of the sapphire disc prior to LM imaging could reduce refractive index mismatch. Another option is to include superresolution LM techniques into the workflow, which are compatible with confocal laser scanning microscopy (Engelmann *et al*., 2014). Therefore our study gives promise to the perspective to obtain correctly registered multimodal image data with high resolution in 3D.

## Supporting information

Disclaimer: Supplementary materials have been peer‐reviewed but not copyedited.

**Fig. S1**. 3D transfer holder. A: Image of the block holder in the loading configuration with different typical resin blocks. B: Technical drawing illustrates the position of the sphere which hold the block sample in a central hole. C–E: Different adapter plates allow defined transfer of the block sample between different instruments. C: Trimming microtome. D: Inverted light microscope E: Electron microscope.
**Fig. S2**. Targeting of test particles embedded in epoxy resin. A: Maximum intensity projection of z‐stack combine confocal fluorescence signal of melamine particles (green channel) and confocal reflection image of block surface (Plan Apochromat 20×/0.8; 140nm pixel^−1^). B: Image of block surface before FIB‐milling is registered to LM image via three corresponding reference marks, which are manually located and highlighted by coloured circles. SEM image was recorded with resolution of 110 nm pixel^−1^. C: Resulting overlay of fluorescence signal allowed prediction of VOI location. Additional to milling window two trenches were applied to mark the boundary of the VOI. D‐I. Series of FIB‐SEM images document the progress of milling through a FITC marked melamine particle (diameter 6μm). As expected the particle was located between the trenches (arrow heads). Scale bar in first panel represents 10 μm
**Fig. S3**. Preservation of ultrastructure. Micrograph and image sequence demonstrate the preservation of ultrastructural details. Virus compartment (VC), mitochondria (M) and nucleus (N). Scale bar represents 1 μm.
