# Supplementary figures and images for: Preservation of protein fluorescence in embedded human dendritic cells for targeted 3D light and electron microscopy
Source: J Microsc. 2015 Mar 18;259(2):121–8. doi: 10.1111/jmi.12230 (PMC4757415; doi:10.1111/jmi.12230)

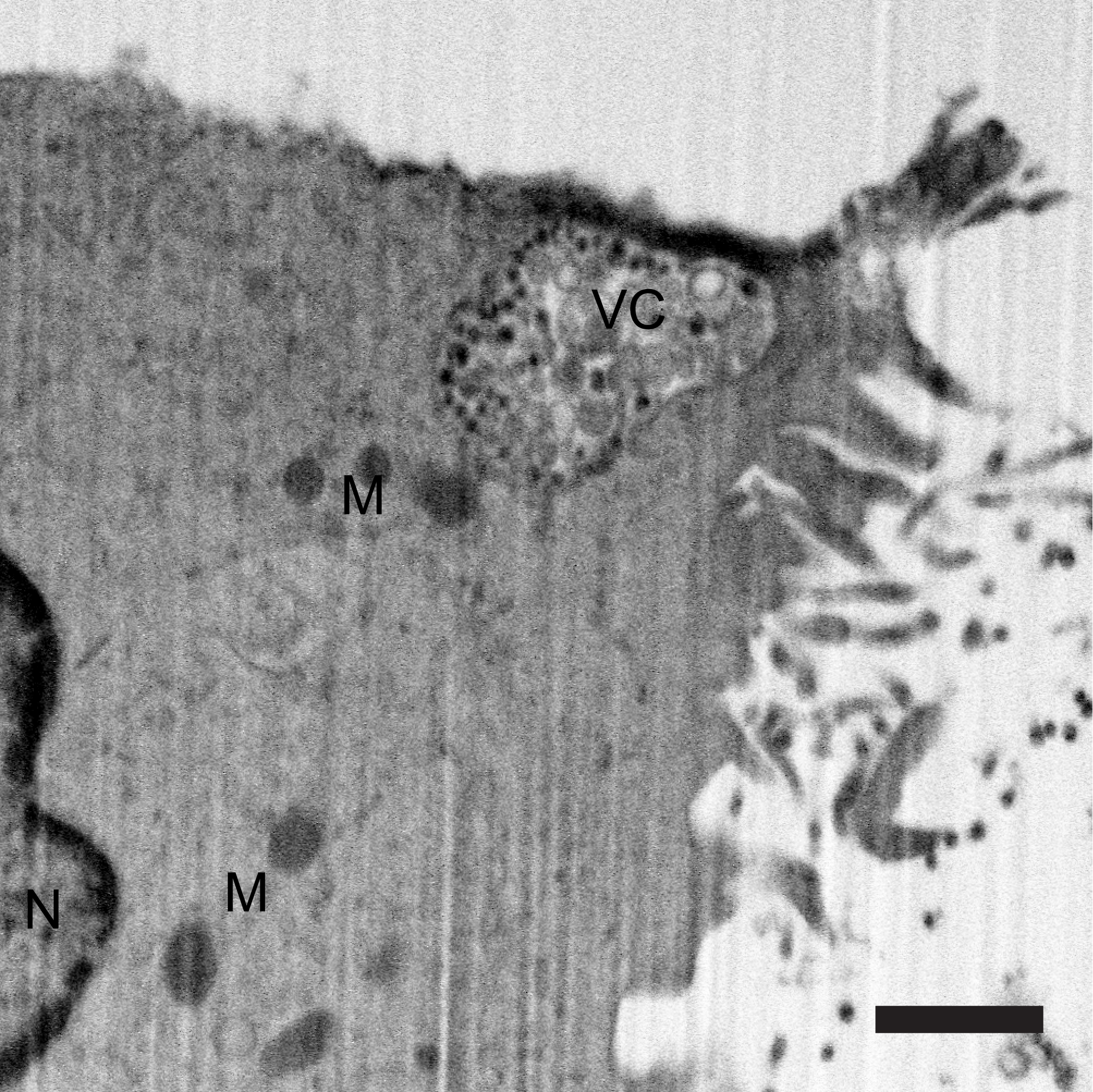

Supplement: Supplementary file 1 — Fig. S1. 3D transfer holder. A: Image of the block holder in the loading configuration with different typical resin blocks. B: Technical drawing illustrates the position of the sphere which hold the block sample in a central hole. C–E: Different adapter plates allow defined transfer of the block sample between different instruments. C: Trimming microtome. D: Inverted light microscope E: Electron microscope. Fig. S2. Targeting of test particles embedded in epoxy resin. A: Maximum intensity projection of z‐stack combine confocal fluorescence signal of melamine particles (green channel) and confocal reflection image of block surface (Plan Apochromat 20×/0.8; 140nm pixel−1). B: Image of block surface before FIB‐milling is registered to LM image via three corresponding reference marks, which are manually located and highlighted by coloured circles. SEM image was recorded with resolution of 110 nm pixel−1. C: Resulting overlay of fluorescence signal allowed prediction of VOI location. Additional to milling window two trenches were applied to mark the boundary of the VOI. D‐I. Series of FIB‐SEM images document the progress of milling through a FITC marked melamine particle (diameter 6μm). As expected the particle was located between the trenches (arrow heads). Scale bar in first panel represents 10 μm Fig. S3. Preservation of ultrastructure. Micrograph and image sequence demonstrate the preservation of ultrastructural details. Virus compartment (VC), mitochondria (M) and nucleus (N). Scale bar represents 1 μm. [file JMI-259-121-s001.zip › jmi12230-sup-0003-Figure S3.tif]

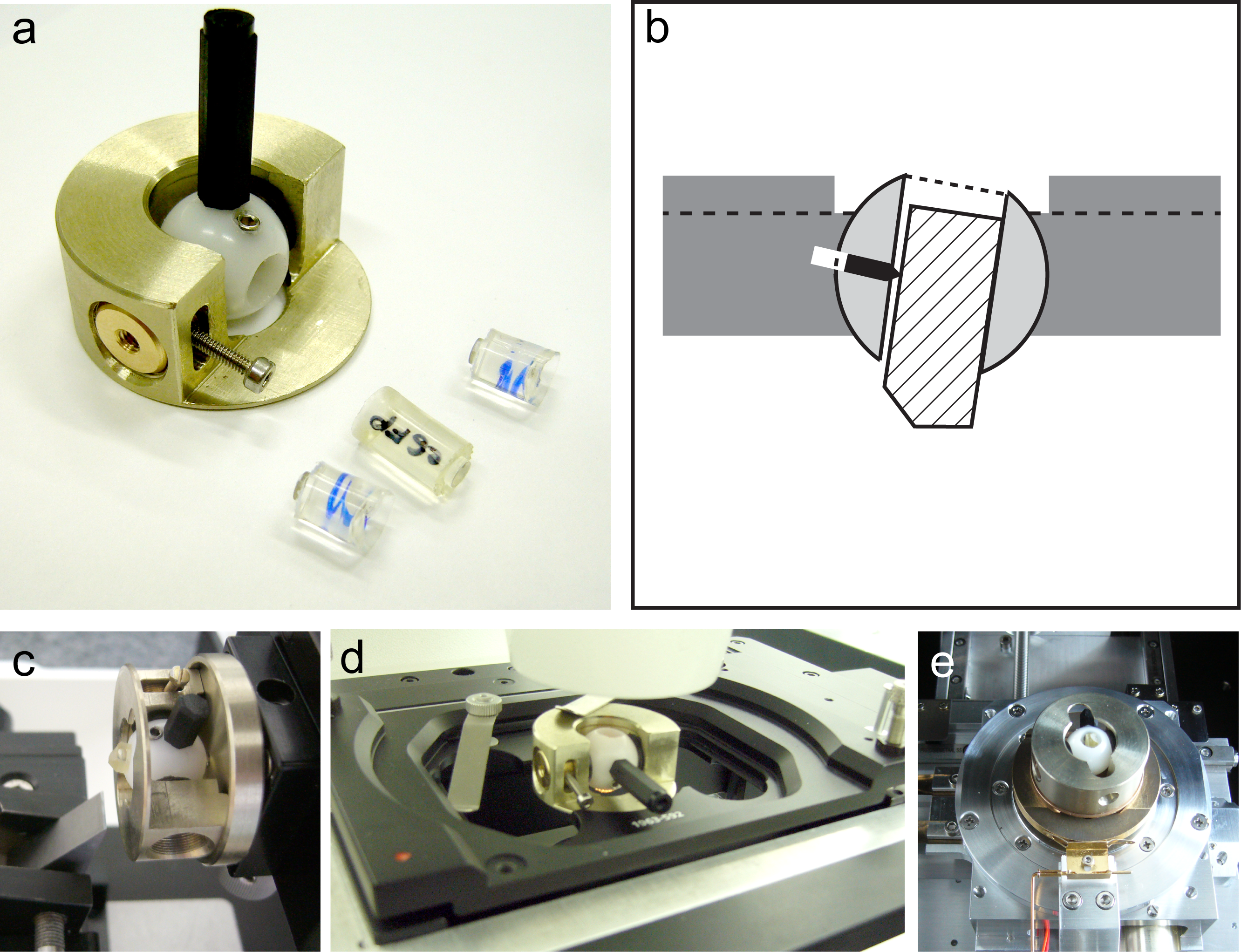

Supplement: Supplementary file 1 — Fig. S1. 3D transfer holder. A: Image of the block holder in the loading configuration with different typical resin blocks. B: Technical drawing illustrates the position of the sphere which hold the block sample in a central hole. C–E: Different adapter plates allow defined transfer of the block sample between different instruments. C: Trimming microtome. D: Inverted light microscope E: Electron microscope. Fig. S2. Targeting of test particles embedded in epoxy resin. A: Maximum intensity projection of z‐stack combine confocal fluorescence signal of melamine particles (green channel) and confocal reflection image of block surface (Plan Apochromat 20×/0.8; 140nm pixel−1). B: Image of block surface before FIB‐milling is registered to LM image via three corresponding reference marks, which are manually located and highlighted by coloured circles. SEM image was recorded with resolution of 110 nm pixel−1. C: Resulting overlay of fluorescence signal allowed prediction of VOI location. Additional to milling window two trenches were applied to mark the boundary of the VOI. D‐I. Series of FIB‐SEM images document the progress of milling through a FITC marked melamine particle (diameter 6μm). As expected the particle was located between the trenches (arrow heads). Scale bar in first panel represents 10 μm Fig. S3. Preservation of ultrastructure. Micrograph and image sequence demonstrate the preservation of ultrastructural details. Virus compartment (VC), mitochondria (M) and nucleus (N). Scale bar represents 1 μm. [file JMI-259-121-s001.zip › jmi12230-sup-0001-Figure S1.tif]

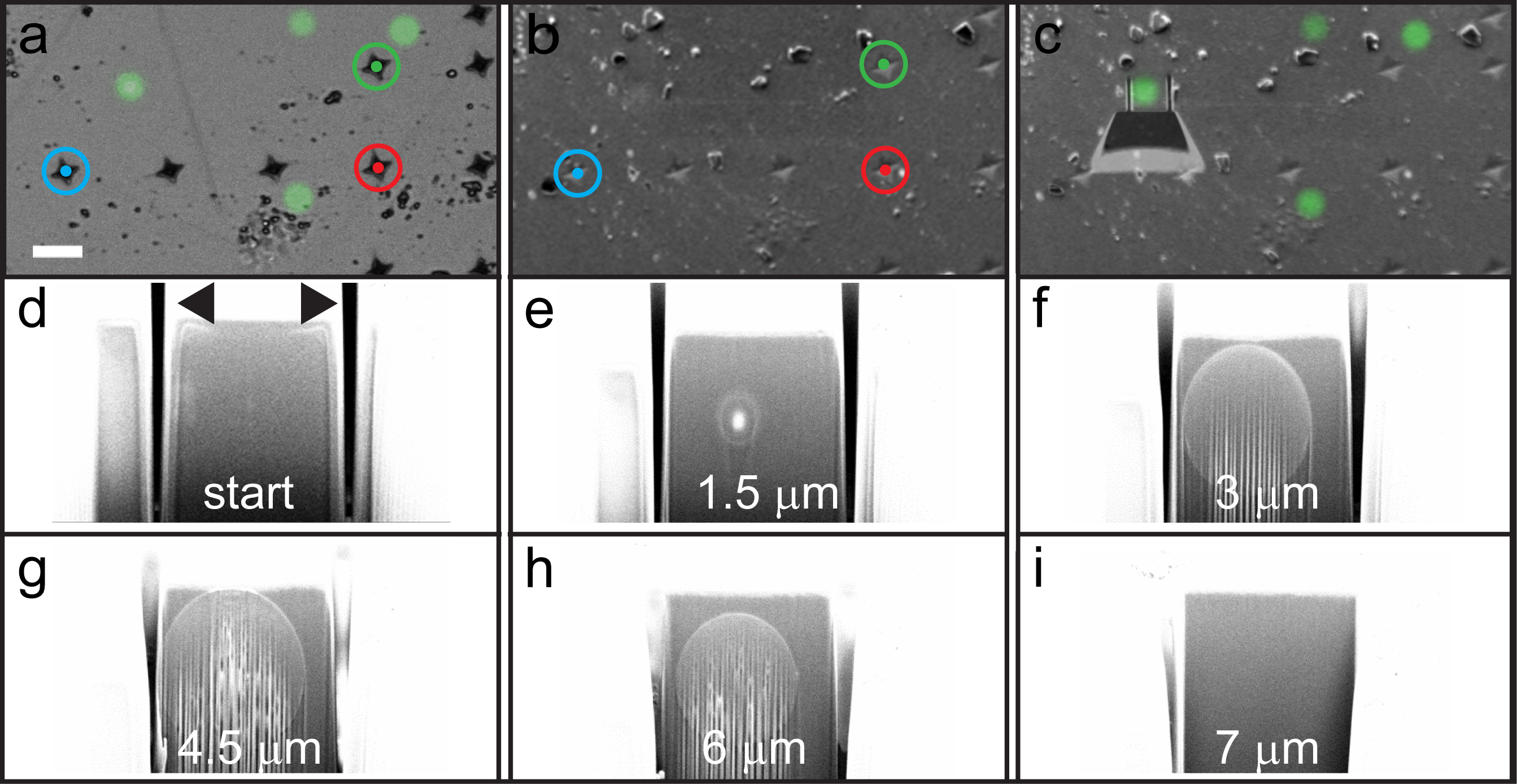

Supplement: Supplementary file 1 — Fig. S1. 3D transfer holder. A: Image of the block holder in the loading configuration with different typical resin blocks. B: Technical drawing illustrates the position of the sphere which hold the block sample in a central hole. C–E: Different adapter plates allow defined transfer of the block sample between different instruments. C: Trimming microtome. D: Inverted light microscope E: Electron microscope. Fig. S2. Targeting of test particles embedded in epoxy resin. A: Maximum intensity projection of z‐stack combine confocal fluorescence signal of melamine particles (green channel) and confocal reflection image of block surface (Plan Apochromat 20×/0.8; 140nm pixel−1). B: Image of block surface before FIB‐milling is registered to LM image via three corresponding reference marks, which are manually located and highlighted by coloured circles. SEM image was recorded with resolution of 110 nm pixel−1. C: Resulting overlay of fluorescence signal allowed prediction of VOI location. Additional to milling window two trenches were applied to mark the boundary of the VOI. D‐I. Series of FIB‐SEM images document the progress of milling through a FITC marked melamine particle (diameter 6μm). As expected the particle was located between the trenches (arrow heads). Scale bar in first panel represents 10 μm Fig. S3. Preservation of ultrastructure. Micrograph and image sequence demonstrate the preservation of ultrastructural details. Virus compartment (VC), mitochondria (M) and nucleus (N). Scale bar represents 1 μm. [file JMI-259-121-s001.zip › jmi12230-sup-0002-Figure S2.tif]
